# A Case of Very Singular Nervous Affection, Supposed to Have Been Occasioned by the Bite of a Tarantula

**Published:** 1808-09

**Authors:** Joseph Comstock

**Affiliations:** South-Kingston, America, (R. I.)


### *A* CASE *of very singular* NERVOUS AFFECTION, *supposed to have been occasioned by the* BITE *of a* TARANTULA. *By Dr.* JOSEPH COMSTOCK, *of South-Kingston, America, (R. I.)*

IN the month of December, 1801, Nancy Hazard, aged 15 years, whose parents lived in North-Kingston, was on a visit upon the Island of Conanicut. The weather in that month being remarkably warm, she went, in company with another young woman, to a stack, to procure some oat-straw to make some female attire for the head. While stooping down and culling the straws which lay on the ground, a large black spider, which she said she observed had very shining black eyes, ran on the back part of her hand, and, without her attempting to molest him, having heard, as she said, that it was good luck, soon ran off. She said it gave her a slight sensation, whilst on the back of her hand, like the prick of a pin, upon a particular part which she pointed out. The same afternoon she felt the hand and arm of that side to twitch involuntarily several times, and that night was taken with a pain in the same, which shifted into her stomach, and increased till the morning of the third day afterwards, when she went into fits.

If I recollect, she puked several times upon the second and third days.

A phy-

A physician was called; but she not having mentioned the spider, and her complaints resembling hysteria, were treated as such by him. This information I received from her and the family. The medicines which he gave, they did not know; she was bled, however.

She not growing any better, Dr. Perry, then my co-partner in medical business, was called. From him I learned that in her case he discovered something very peculiar, and was led to consider it a case *sui generis*.

He observed that her fits resembled, in some measure, hysteria, but more nearly hysteric paroxysms combined with the St. Vitus's dance.

After premising a gentle cathartic, he gave her the tinct. castor, spt. C. C. and an opiate at night. By these it was thought some relief was afforded; but the fits returned the next day, and were very obstinate and distressing.

Being at the distance of twelve miles from her, he did not visit her again till five or six days afterwards. Her situation was not essentially altered: the fits continued to afflict her the greater or less part of every day. They were not accompanied with fever. To the former remedies he joined the oleum succini, with orders to increase the dose of the opiate.

The Doctor, after this visit, first mentioned to me an extraodinary increase of sensibility, which he observed in her feeling, and which constituted, afterwards, a prominent and very striking feature in her complaint.

There had no mention been made by her, as yet, respecting the spider; but that she had an inclination for music was judged by her movements with her hands and fingers, when in her fits, as if after a tune: and when music was introduced, her motions corresponded; she beating with her hands and finger ends upon her breast, in imitation of dancing. And sometimes, when, from the violence of the spasms, she could not regulate her motions, and would strike too hard, it was common for some of the attendants to place their hand upon that part of the thorax where the ends of her fingers struck, to prevent the harm. She appeared best pleased when her father's hand was placed for this purpose; and if another person ever so gently endeavoured to displace his hand, and put his own in its place, she would immedately push away the strange hand, and seek for her father's! This she did, although to every thing else insensible, and with her eyes closed.

This

This was not done by taking the different hands into her own, and discriminating them by their magnitude, softness or hardness; for this the violence of the spasm, with which she was universally affected, rendered her incapable of doing; but simply by touching with her finger ends, as the Doctor experienced in himself. The violence and long continuance of the paroxysms having induced debility, some tonic remedies were added to the above; after which she was removed home, appearing somewhat better, or having only, perhaps, a longer interval of ease.

The disorder, however, soon returned with increased violence; and the medicines, after repeated trials, and increased doses, not appearing to have any effect, were at length discontinued, from a conviction of both physicians and friends that they were of no utility. Music now became the substitute for medicine, and was absolutely necessary, it being the only thing which moderated the violent and distressing spasms with which she was the greatest part of the time afflicted.

It was the fortieth day of her illness when I first visited her. When I went into the room she was sitting in a chair, leaning her head upon one of her friends, and following, with motions of her hands, a tune sung by some young woman who appeared to attend for that purpose. The tune she followed with the greatest regularity and exactness. If it changed for an air more quick or slow, her motions exactly corresponded; if it stopped, her motion immediately ceased. Yet to all other sounds did she appear as insensible as matter inanimate.

Her muscles were continually affected with spasm during the paroxysms. Before the music began, or if it ceased, the spasms were very violent and excruciating, affecting her muscles with convulsive motions. When the music commenced, it in a great degree relaxed the spasmodic tension, and the convulsive motions were changed for the motion of her hands in following the tune. The tension remained, however, in her fingers, which, all the time of the fit, were drawn apart as far as possible, and rendered stiff as sticks. She did not now, as she did afterwards, dance upon her feet.

After remaining in this situation about an hour the fit began to abate. She was now able to regulate the motions of her hands, and to speak, though indistinctly.

She was in this situation when I was desired to approach her, that I might witness something still more extraordinary nary than a disorder for which music was the only remedy yet found out.

Her eyes had not been open since I came into the room; and, to my surprise, without opening them, and by feeling only, she accurately told the colour of the different garments I had on!

This faculty she possessed in such an eminent degree as to be able to point out different colours upon one piece of cloth, cotton, linen or woollen, and to be able to discover different kinds of materials when wove into one piece, as woollen, silk and cotton. More exquisite still was this sensation: After feeling my hand some minutes with great attention, she would afterwards tell what things received from my hand belonged to me. This cost her more time and study than the other; and I observed that, in order to determine this, she put those substances on her cheek, as she had done my hand before them.

Did holding a substance in one's hand make a slight alteration in its surface, which her extreme sensibility of feeling could discover? And having before felt that hand, could she perceive the similarity? I was led so to conclude, or to leave the phenomena unaccounted for.

I was strengthened in this opinion by what further happened. I held a piece of money some time in my own hand, and then gave it to another person to hand to her. This appeared to puzzle her very much; and after feeling of it for some time with great attention, she accidentally let it drop, and then said she believed that it belonged to the person who passed it from me to her; but upon its being put into her hand again, she put it to her cheek, and determined that it was mine. Perhaps it will be said that by my own reasoning she ought to have adjudged it to the person who last held it: but I must remark that I held the piece longer than the other person, who, as he passed it from me to her, only slightly touched it. If, therefore, holding it in one's hand altered the surface in a slight degree, it was probably altered most by my hand. Her putting it on her cheek was probably owing to the sense of feeling being more acute there than in her fingers, she having been accustomed to use her fingers when in health. I have observed persons to do the same when they wished to perceive a slight degree of warmth in any body, or the smoothness or softness of any material.

I should not relate things so much surpassing common credibility as those I have related, and am hereafter, in this account, to relate, let my senses confirm them ever so so unequivocally, out of sight of credible witnesses. But these, and the other circumstances which I shall mention, were all observed in open day, and in the presence of persons whose testimony is not to be doubted.

All this time she was partly in a fit. She soon after came entirely out of it; was rational as when in health; and all this magic sensibility vanished; she remembering nothing of what had happened during the paroxysm.

I was now led to consider her disorder a real *tarantismus;* and upon inquiry, she related the history of the spider, and the affection of her arm the same day, as above related. The partiality which she had formerly shown, when in her fits, for her father, had now begun to abate, and even to be changed to an aversion: but she still evinced a peculiar regard for her relations more remote, as cousins, &c.

Upon the back of the hand of one of those she chose to play with her fingers in following the music; and I now made the same experiment which Dr. Perry had done early in her illness. I put my hand, at the instant that both her's were lifted up, in the place of her relation's. The consequence was as before: immediately upon the ends of her fingers touching it, she endeavoured to remove it, and felt for the other!

I did not see her again until the twelfth week from the commencement of her illness, but received frequent intelligence from her. Her fits returned at unequal periods during that time. Sometimes these intervals were protracted two or three days, in which she enjoyed good health, which was usually the case in the absence of the fits; some irregularity of the menstrua only excepted.

The reports spread about the country of her strange illness, and surprising faculty of distinguishing colours by the touch, which some vulgarly attributed to witchcraft, drew together a great many people to see her. The throng of spectators, and the extreme attention she was obliged to make use of in order to distinguish colours, appearing to fatigue her, her parents were advised to prevent her being put upon the trial, which was accordingly done. I did not now put her upon any trial of this kind; but the sensation appeared to continue as acute as ever; for she readily distinguished her relations by the touch; to all of whom she now manifested as great an aversion as she had before shown fondness. None of them were able to touch her, when in her fits, without aggravating every symptom, and bringing on the most violent spasms. And it was even said said she could ill bear the room in which her father was, and could tell if he was in the room, though ever so dark, by the effect it had upon her disorder.

The salutary effect of music still continued; and she now danced upon her feet with as much accuracy as she had before shown in following the tune with her hands. Her propensity for this was discovered by her movements with her feet as she lay on the bed.

It was in the fore part of the day when I called to see her. She lay in a motionless state upon the bed, every nerve and muscle being contracted. In this situation she had been from the early part of the preceding evening; not having spoken, nor taken any notice of the music, which was now made by an excellent performer on the violin, singing having before this time lost its influence upon her. It was only of music of this kind that she would take any notice.

He had, in vain, tried to rouse her during the night. I desired him to make a further trial, by putting the instrument near her ear, and playing a brisk air. This had the desired effect. I soon observed her fingers, and then her eye-lids, to relax. As she opened her eyes I discovered nothing but the white; the whole cornea, or coloured part, was turned over upwards into the socket, by the violence of the spasm upon the muscles of those parts.

She then began to follow the tune with her fingers, and shortly after came out of the fit, got up, walked about, and adjusted her dress. We remained out of the room a few minutes; when we went in she had thrown herself upon the bed, and was in the same motionless, contracted state, as when I first came in.

She remained thus until the violin was brought. After the music began, she made motions first with her hands and then with her feet. This was the signal for helping her up. A young man, not one of the family, but a distant relation, happening that instant to enter, attempted to assist her, but the moment he touched her she shrunk back with a seeming horror, although her eyes were closed, and would have gone into violent convulsions, if he had not desisted, by desire of the musician. I not being related, helped her on her feet. She soon commenced dancing, in which exercise she remained near an hour and a half, with very little intermission. She danced with all her might, jumping a considerable distance from the floor, and continued the exercise so long that, being entirely exhausted, she would fall without attempting to prevent it, like a log upon the the floor. After panting with great emotion a few seconds, she would resume the exercise.

After having kept her dancing till she refused the exercise any longer, she was again laid on the bed, and fell into the same state of stupor as before.

Whilst dancing, her eyes all the time were closed, which appeared to proceed from the violence of the spasms; as to me, indeed, did all her motions. The conjecture may be too bold, but I will venture to suggest it,—that those spasmodic affections which were so violent were brought into a system of regularity by the power of music upon the auditory nerves, and sympathetically communicated from them to the other nerves of the body; and thus was her whole dancing spasmodic and involuntary.

One circumstance, among others, which corroborates this opinion, is, that in a very violent paroxysm, when the music would for some time lose its effect, and her spasms were so excessive, that her friends were apprehensive they would prove fatal, when first the music would make an impression, it would cause the motions of her body, in dancing, to be as extravagant as the spasms had been violent.

No one would pretend that the spasms which preceded her dancing, and into which she would fall in case the music ceased, were dependant upon the will. And it appeared very evident that her dancing, was nothing more nor less than a modification of those spasms, one terminating in the other, and *vice versa*.

When I left her, I recommended to her friends to keep her dancing as long as possible when she once began, and to trust to it as a principal remedy. It was my opinion that nature in this way pointed out relief.

I was afterwards informed that this advice was followed, and it was attended with this effect: it protracted the space between her fits to a longer period than ever before it had been. When they returned they were as severe as ever, though the interval was lengthened from days to weeks.

The next time I saw her I observed a surprising acuteness in her olfactory nerves. She had manifested a partiality for the smell of certain odoriferous substances. Essence of lemons was one of those she liked best. She was in a partial paroxysm when a vial of it was introduced, and held towards her. She drew in the scent by an incessant and convulsive kind of snuffing. It was afterwards carried out of the room, and she, with her eyes shut, directed her head toward the passage the person went went out at, still snuffing. Before it was carried out, the musician rubbed the end of his finger around the mouth of the vial. He did not turn it up, and there was not the least particle of the essence, in a fluid state, upon that part. There was the remains of the oil, having a yellowish appearance, the thin part being entirely exhaled.

After the vial was removed, he put his finger under the bed on which she was lying, about half way between it and the floor. She instantly turned over on her face, applying it to the bed, that she might be able to inhale the beloved odour in a right line, which she did with the greatest eagerness. As she emerged still more from the fit, so as to open her eyes, she discovered a partiality for certain colours, and an aversion to others. Green and red were among the former; white and black, and their mixtures, among the latter. If the agreeable colour was presented, and moved or shook in her sight, it appeared to produce the greatest pleasure, which she evinced by a convulsive kind of laughter, very hearty, yet not natural. If white or black was presented, she would turn away her head with a marked aversion and seeming horror. The sight of any thing transparent, and water slowly poured from one vessel into another, produced the same effect as her favourite colours.

After this visit I frequently called to see her, as my professional avocations led me into her neighbourhood.

Every new paroxysm was attended with some variation; but the most striking phenomena were a repetition of what I have already related: the others were referable to the same cause in a less degree, that is, *increased sensibility.*

She continued to have her fits return at irregular periods, sometimes with intervals of four or five weeks, until the month of August, 1802. She was then seized, whilst from home, with a pain in that hand upon which the spider had crawled the December before; and the spot where she said the spider had bitten her soon became red. The pain increased for two or three days, when a spot, as large, or a little larger than half a dollar, began to turn black, and threatened gangrene. Dr. Perry, who happened to be in her neighbourhood at this time, called to see her, and ordered the cortex ruber in substance. By the use of this, and some simple topical applications, the mortified part cast off, leaving a spot of an irregular shape, very much resembling a burn.

 During

During the soreness of her hand she was again seized with fits, but they were not so violent as formerly.

When I saw her, which was, I think, on the fourteenth of the month, she lay in a state of stupor similar to that I mentioned above.

The people about her remarked that her hearing was very acute, and that she had been known to understand the conversation of persons in a whisper, though in another room. I did not witness any instance of this; but the lady who mentioned it to me, related it from her own knowledge, and is of the first respectability, and unquestionable veracity. The young woman was then at her house, and from other instances of the extreme sensibility of her nerves, which I witnessed myself, and the aptitude I observed in that sensibility to change from one set of organs to another, I make no doubt of the fact.

Her hand, soon after this period, began to discharge a very unusual kind of matter. It was as green, and almost as thick as inspissated beef gall. This matter was discharged in such quantities, for two or three weeks, as to require the sore to be dressed three times a day; and although several folds of linen were laid over the dressings, they would be soaked through before the next dressing, and a large quantity of matter collected on her hand. The fits abated when the discharge began, and she has since had no return of them, and nearly five months have now elapsed. The discharge gradually lessened, and the sore healed. Once during this period the discharge suddenly stopped, upon which she grew very unwell, and was threatened with fits, but upon its re-appearing she grew better.

She is a girl of good habit of body, never having been subject to any particular disease, and, in general, very healthy.

Her temperament I should place in the *sanguineo-choleric.*

It was observed by the family, that during the discharge from her hand, the arm of that side was considerably diminished in size.

I never observed her complaints accompanied with any pyrexia, nor was her flesh, during her illness, diminished in any considerable degree.

I once saw her in a partial paroxysm, when she appeared very sick at her stomach, and kept continually retching. I [illegible]rdered some warm water to be given her, but she did not puke it up; and what she evacuated did not appear to come come from her stomach. It was a mucous matter, appearing to come from the mucous follicles about the fauces, and I conclude, an increased secretion of that fluid, as she evacuated considerable quantities of it. It was thus that she threw up pins and needles, which, in the agony attending her fits, she would, I conceive, unwittingly put in her mouth, and in this way get rid of them; which gave rise to a vulgar report, that she swallowed those substances and then puked them up.

A case so replete with the wonderful is not, perhaps, easily to be found in the annals of medicine; and it is to be wished that a more accurate account had been kept of it. But from the length of time she was sick, and from the circumstance of no one physician attending her constantly, and a great part of the time none at all, this was impracticable.

I have endeavoured to collect the leading and most prominent symptoms, and rather to lessen than exaggerate those which partook so much of the marvellous.

In relating thus much, I may, perhaps, be charged with presumption by some; but I have no fear of this from those who will take the trouble to inquire into the numbers and credibility of those who witnessed every part of my narrative.

Respecting the insect which I have supposed to be the cause, it seems natural to make some remarks. It may be objected that the tarantula (phalangium apulianum) is not an inhabitant of our climate. And this objection, so far as I know, is well founded; that being a name given to a particular species of spider in Italy. But I think we have a spider in this country, whose economy is somewhat similar to the description given of that; and, if it be not the same species, may be equally poisonous. I will not contend about names. That she was poisoned by a spider, considering every circumstance, will seem hard to deny. She was heard to say, by a person near her, at the time she was at the stack, "O, what a great spider there is!" And then the first complaint being in that arm, and ending in the same, and a sore of an unusual kind appearing in the very spot where she all along had said the spider had bitten her, are strong proofs of her having been bitten; not to mention that such a strange illness would naturally lead us to look for an unusual cause. In some of the symptoms we observe a similarity to those occasioned by the Italian spider, but here we cannot trace a parallel clearly through. The spider of Italy produces a disorder, which, as their physicians say, is cured by music. Here the parallel fails. In this case the patient was evidently cured by a spontaneous discharge of matter at the very spot where we supposed the poison first entered the blood. In respect to those remarkable effects, the consequense of increased sensibility, we have no parallel that I know of. In respect to the partiality for, and antipathy to certain colours, the parallel is exact in the two countries. In respect to music being the cause of the patient's dancing, and also in its affording relief to the complaint, there is an exact similarity between this case and those described by the Italians. In respect to the partiality shown for her kindred at one time, and the remarkable aversion evinced at another, I know of no parallel.

The lateness of the season in which I have supposed the spider to have appeared has not been offered as any objection to its appearance; nor will that be adduced by any one who recollects the extreme mildness of the fall months, and the month of December that year. It was observed to me by the father of the young woman, that the warmth of the day, on which his daughter went to the stack was such, that she and another young woman took off their outside garments, and sat in the door to work. It may be proper to remark, that when I first saw her I could perceive no mark upon the spot where she supposed the spider had bitten her. This was, however, as late as the fortieth day; and if it had been earlier examined, it is not very probable that any very perceptible mark from an insect which inflicts a wound in the manner of the tarantula could have been discerned; it not being by biting, but by pricking with a very fine pair of horns or forceps, and, at the same instant, spitting from its mouth the poisonous fluid upon the wound: therefore it cannot appear strange that the puncture should be scarcely, if at all, visible at first.

I consider the remarkable increase of sensibility as constituting a part of the disease; she never showing any signs of it when she was entirely out of her fits.

Perhaps this case may furnish us with some new light relative to the treatment of nervous diseases. If my theory be right, may we not expect much benefit from music in hysteria?

I have had one case only in which I had an opportunity of trying it: it was an obstinate hysteric one. It was attended with the advantage I expected. It moderated the spasms, reduced them to order, and shortened the paroxysm.

*January 24,* 1803.

